# Cost of illness and program of dengue: A systematic review

**DOI:** 10.1371/journal.pone.0211401

**Published:** 2019-02-20

**Authors:** Luana Nice da Silva Oliveira, Alexander Itria, Erika Coutinho Lima

**Affiliations:** 1 Faculty of Pharmacy, Center for Economics and Health Assessments, Institute of Health Technology Assessment, Federal University of Goiás, Goiás, Brazil; 2 Institute of Tropical Pathology and Public Health, Department of Collective Health, Federal University of Goiás, Goiás, Brazil; 3 Center of Economics and Health Assessments, Institute of Health Technology Assessment, Federal University of Goiás, Goiás, Brazil; 4 Faculty of Pharmacy, Federal University of Goiás, Goiás, Brazil; Public Health England, UNITED KINGDOM

## Abstract

**Background:**

Studies on dengue related to the cost of illness and cost of the program are factors to describe the economic burden of dengue, a neglected disease that has global importance in public health. These studies are often used by health managers in optimizing financial resources. A systematic review of studies estimating the cost of dengue was carried out, comparing the costs between the studies and examining the cost drivers regarding the methodological choices.

**Methods:**

This study was done according to the guidelines of the Centre for Reviews and Dissemination (CRD). Several databases were searched: Medline, Virtual Health Library and CRD. Two researchers, working independently, selected the studies and extracted the data. The quality of the methodology of the individual studies was achieved by a checklist of 29 items based on protocols proposed by the British Medical Journal and Consolidated Health Economic Evaluation Reporting Standards. A qualitative and quantitative narrative synthesis was performed.

**Results:**

A literature search yielded 665 publications. Of these, 22 studies are in accordance with previously established inclusion criteria. The cost estimates were compared amongst the studies, highlighting the study design, included population and comparators used (study methodology). The component costs included in the economic evaluation were based on direct and indirect costs, wherein twelve studies included both costs, twelve studies adopted the societal perspective and ten studies used the perspective of the public health service provider, or of a private budget holder.

**Conclusion:**

This study showed that the cost of dengue in 18 countries generated approximately US$ 3.3 billion Purchasing Power Parity (PPP) in 2015. This confirms that the burden of dengue has a great economic impact on countries with common socioeconomic characteristics and similarities in health systems, particularly developing countries, indicating a need for further studies in these countries.

## Introduction

Dengue is a systemic viral disease, and the main vector of epidemiological importance in the transmission of dengue virus (DENV) is *Aedes aegypti* [1].

A disease of global importance in public health affecting more than 100 tropical and subtropical countries. Recently there have been reports of epidemics in non-endemic areas of Europe and the United States where transmitter mosquitoes have possibly settled through infected travelers, enabling transmission cycles [2].

A recent study estimates there to be 390 million dengue infections every year, of which 96 million have clinical manifestations. The World Health Organization (WHO) estimated about 3.2 billion people worldwide in 2015 were in the probability of catching the disease [3,4].

The increase in cases of dengue fever has made this disease an issue for society, but specifically for the health authorities due to the difficulties to control the epidemic caused by the dengue virus and insufficient health services to care for the affected population [5].

In this scenario, epidemiological surveillance has been an important tool for decision- making, aiming to provide useful evidence to enable decision-makers in health to lead and manage dengue cases/ policies more effectively [6].

The Objective of the study was to understand the current state of the art for both cost of illness and program studies, through a systematic review, which is important to support economic evaluations.

## Methods

### Study design

A systematic review of dengue cost analysis studies was carried out. It was developed by 2 researchers from the Faculty of Pharmacy (FF) of the Federal University of Goiás (UFG). The guiding question of the systematic review was: What is the cost of the dengue program and disease?

### Search strategy and article selection

A search of the studies was conducted in the following databases: Medline (via PubMed), Virtual Health Library (VHL), The Cochrane Library and Center for Reviews and Dissemination (CRD). Only studies in English, Spanish and Portuguese were selected according to the Methodological Guideline: elaboration of a systematic review and meta-analysis of randomized clinical trials, as an attempt to increase the reproducibility of the study, since there is no guideline for systematic review in economic studies. [7].

A survey of the studies was carried out starting from 2005, due to a large number of dengue epidemics registered that year with a significant increase of serious cases and deaths in Brazil [8]. In addition, a systematic collection of publications mainly related to costs was made based on the last ten years [9]. The timeline and search strategy are shown in Table 1.

**Table 1:** Research strategy for the systematic review.

Database	Search strategies	Timeline
MEDLINE (via PubMed)	"dengue"[MeSH Terms] OR "dengue"[All Fields]) AND	02 October 15
	("economics"[Subheading] OR "economics"[All Fields] OR "cost"[All Fields] OR "costs and cost analysis"[MeSH Terms] OR ("costs"[All Fields] AND "cost"[All Fields] AND "analysis"[All Fields]) OR "costs and cost analysis"[All Fields]) AND programme[All Fields]”	
	("dengue"[MeSH Terms] OR "dengue"[All Fields]) AND	
	("economics"[Subheading] OR "economics"[All Fields] OR "cost"[All Fields] OR "costs and cost analysis"[MeSH Terms] OR ("costs"[All Fields] AND "cost"[All Fields] AND "analysis"[All Fields]) OR "costs and cost analysis"[All Fields]) AND ("disease"[MeSH Terms] OR "disease"[All Fields] OR "diseases"[All Fields]) OR ("illness"[MeSH Terms] OR " illness "[All Fields] OR " illness "[All Fields])	
VHL	dengue AND cost AND program OR disease OR illness	21 June 16
CRD	(dengue) AND (program) OR (disease) OR (illness)OR (cost)	21 June 16

VHL: Virtual Health Library; CRD: Centre for Reviews and Dissemination.

After the search of the studies in the databases, a screening was conducted by reading the titles and abstracts, performed by the review team independently.

Searching for studies in all possible sources of data generates a much larger number of articles than would actually qualify for the established criteria. This is because the search strategy is elaborated by ensuring sensitivity over specificity. Thus, for the screening of studies, the sum of the total number of articles in all databases is recorded and the title is quickly read, allowing the selection of references and discarding a large number of references that do not fall under the eligibility criteria established by the Commission Review [7].

A free reference manager software, Mendeley, was used for sorting the articles, accounting of duplicates, organization of references, practicality and for optimization of time. The studies that went through the screening had their full text recovered. The eligibility of the studies was confirmed after reading the full text and selecting observational studies (case-control, cohort and cross-sectional) that presented economic evaluations, costs of dengue, program or illness, considered populations at risk for dengue disease, had no limit of sex, race or age, and the outcome in unit monetary policy. We excluded studies that presented proposals for new prevention measures, assuming the cost that could generate (S1 Table).

The selection of the studies and the screening were performed independently by two researchers and the results were compared. Disagreements in 15% of the documents were resolved in consensus meetings by arbitration through a third party investigator, when necessary.

### Data extraction

The two researchers, who independently assessed the compliance of the full texts with the inclusion criteria, knew the names of the authors, institutions, year and scientific journals when they applied the eligibility criteria.

A data extraction form was prepared and was used for this purpose. The form was divided into 3 sections, according to the types of information provided by the studies:

- Section A—General information about selected studies (Table 2).

**Table 2:** Section A- general information about selected studies.

First author/ Year publication	Cost analysis (illness / program)	Sources of funding	What sources of funding
Adriana Rodríguez, 2012 [14]	Cost of illness	No	-
Alessandra A. Machaof, 2014 [15]	Cost of illness	No	-
Blas Armien, 2008 [16]	Cost of illness/ program	Yes	Pediatric Dengue Vaccine Initiative (PDVI)
Carlos A. R. Pereira, 2014 [17]	Cost of illness	No	-
Donald S. Shepard, 2011 [18]	Cost of illness	Yes	Sanofi Pasteur
Donald S. Shepard, 2012 [19]	cost of illness	Yes	Sanofi Pasteur
Donald S. Shepard, 2014 [20]	Cost of illness	Yes	Sanofi Pasteur
Donald S. Shepard, 2016 [21]	Cost of illness	Yes	Sanofi Pasteur
Eduardo A. Undurraga, 2015 [22]	Cost of illness / program	Yes	Sanofi Pasteur and Brandeis University and was also partially supports of the UBS Optimus Foundation
Frances E. Edillo, 2015 [23]	Cost of illness	Yes	Sanofi Pasteur, The Global Emerging Infection Surveillance and Response System
Frederic W. Selck, 2014 [24]	cost of illness	No	-
Hans-Christian Stahl, 2013 [25]	Cost of illness/ program	No	-
Helena Taliberti, 2010 [26]	Cost of illness	No	-
Jose A. Suaya, 2009 [27]	Cost of program	Yes	Pediatric Dengue Vaccine Initiative (PDVI)
Julien Beauté, 2010 [28]	Cost of illness /program	No	-
Neil Thalagala, 2016 [29]	Cost of illness/ program	Yes	International Research Consortium on Dengue Risk Assessment, Management, and Surveillance
Pankaj Garg, 2008 [30]	Cost of illness	No	-
Pham Thi Tam, 2012 [31]	Cost of illness	Yes	Australian Non-Government Organisation Cooperation Program
Raúl C. Rodríguez, 2016 [32]	Cost of illness	Yes	Sanofi Pasteur
Sandra M. Santos, 2015 [33]	Cost of program	Yes	National Council for Scientific and Technological Development (CNPq)/ Ministry of Science, Technology and Innovation(MICT)
Sonia Tarragona, 2012 [34]	Cost of illness	No	-
Uhart M., 2016 [35]	Cost of illness	Yes	Sanofi Pasteur

- Section B—Information on study design, population included and comparators used (study methodology) (Table 3).

**Table 3:** Section B—Information on study population included and comparators used (study methodology).

First author/ Year publication	Study population	Cost components included in the economical evaluation	Study period	Method of collecting cost data (gross or micro)
Adriana Rodríguez, 2012 [14]	Santiago de Cuba	Direct and indirect costs (Hospitalization and Ambulatory)	2006–2007	Micro costing
Alessandra A., 2014 [15]	Brazil	Direct costs (Hospitalization)	2010	Micro costing
Blas Armie, 2008 [16]	Panama	Direct and indirect costs (hospitalization/ Ambulatory)	2005	Gross costing
Carlos A. R. Pereira, 2014 [17]	Brazil	Direct and indirect costs (Hospitalization and Ambulatory)	2011	Gross costing
Donald S. Shepard, 2011 [18]	North America, Central America and Mexico, the Andean region, Brazil, the Southern Coneand the Caribbean region	Direct and indirect costs (Hospitalization and Ambulatory)	2000–2007	Micro costing
Donald S. Shepard, 2012 [19]	Sri Lanka	Direct and indirect costs (Hospitalization and Ambulatory)	2009	Gross costing
Donald S. Shepard, 2014 [20]	India	Direct costs (Hospitalization and Ambulatory)	2006–2012	Gross costing
Donald S. Shepard, 2016 [21]	World	Direct and indirect costs	2013	Gross costing
Eduardo A. Undurraga, 2015 [22]	Mexico	Direct costs (disease and vector control)	2010–2011	Micro costing
Frances E. Edillo, 2015 [23]	Philippines	Ambulatory public and private costs, public and private hospital costs of DF and DHF, total cost	2008–2012	Micro costing
Frederic W. Selck, 2014 [24]	World	Direct and indirect costs (Hospitalization and Ambulatory)	2011	Gross costing
Hans-Christian Stahl, 2013 [25]	Peru, The Dominican Republic, Vietnam and Indonesia	Direct and indirect costs (Hospitalization, Ambulatory, vector control)	2011	Gross costing
Helena Taliberti, 2010 [26]	Brazil	Direct costs (vector control)	2005	Gross costing
Jose A. Suaya, 2009 [27]	Americas and Asia	Direct and indirect costs (Hospitalization and Ambulatory)	2005	Gross costing
Julien Beauté, 2010 [28]	Cambodia	Direct costs, Dalys	2006–2008	Micro costing
Neil Thalagala, 2016 [29]	Sri Lanka	Direct cost vector control and direct costs (hospitalization)	2010–2012	Micro costing
Pankaj Garg, 2008 [30]	India	Direct costs (Hospitalization)	2006	Micro costing
Pham Thi Tam, 2012 [31]	Vietnam	Direct and indirect costs (Hospitalization and Ambulatory)	2006–2007	Gross costing
Raúl C. Rodríguez, 2016 [32]	Colombia	Direct and indirect costs (Hospitalization and Ambulatory)	2010–2012	Gross costing
Sandra M. Santos, 2015 [33]	Brazil	Direct costs (vector control)	2009–2010	Micro costing
Sonia Tarragona, 2012 [34]	Argentina	Direct and indirect costs (Hospitalization and Ambulatory)	2009	Gross costing
Uhart M., 2016 [35]	District of France	Direct costs (Hospitalization)	2007–2011	Gross costing

- Section C—Information from the perspective of the study, and addition of cost result in dollar PPP in the year 2015 (Table 4).

**Table 4:** Section C—Information on costs, specifying the types of costs (study outcome).

First author/ Year publication	Study perspective	Conversion PPP dollars (2015)
Adriana Rodríguez, 2012 [12]	The public health service provider	17,46 million
Alessandra A. Machaof, 2014 [13]	The public health service provider	286.52 thousand
Blas Armien, 2008 [14]	The public health service provider	42.2 million
Carlos A. R. Pereira, 2014 [15]	Society	44.29 thousand
Donald S. Shepard, 2012 [17]	Society	162.04 million
Donald S. Shepard, 2014 [18]	The public health service provider	2.16 billion
Eduardo A. Undurraga, 2015 [20]	Society	33.4 million
Frances E. Edillo, 2015 [21]	The public health service provider	151.62 million
Hans-Christian Stahl, 2013 [23]	Society	Vietnam 7.57 million
Helena Taliberti, 2010	Payer perspective	Indonesia 4.05 million
Jose A. Suaya, 2009	Society	Peru 1.53 million
Julien Beauté, 2010	Society	Dominican Republic 20.47 million
Helena Taliberti, 2010 [24]	The public health service provider	20,82 million
Julien Beauté, 2010 [26]	The public health service provider	15,27 million
Neil Thalagala, 2016 [27]	The public health service provider	13.61 million
Pankaj Garg, 2008 [28]	Society	132.58 million
Pham Thi Tam, 2012 [29]	Society	82.9/ per patient
Raúl C. Rodríguez, 2016 [30]	Society	216.25 million
Sandra M. Santos, 2015 [31]	The public health service provider	551.01
Sonia Tarragona, 2012 [32]	Society	11.59 million
Uhart M., 2016 [33]	Payer perspective	7.87 million

The data was extracted and arranged into tables in the excel program in a standardized and methodological way to allow specification of the cost informed. This grouping was performed to facilitate the comparative analysis of the studies, favoring the identification of the variability between them.

### Quality assessment

The quality of a systematic review depends on the validity of the studies included in it, so at this stage of the quality assessment of each study it is important to consider all possible sources of error (bias) in order to generate results that may be reliable [10].

For health cost analysis, a checklist was developed based on the protocol proposed by the British Medical Journal (BMJ), and on the Consolidated Health Economic Evaluation Reporting Standards (CHEERS) in 2013 by the International Society for Pharmacology and Outcomes Research (ISPOR) [11,12].

The BMJ checklist allows to evaluate the items of a health economic evaluation, while the CHEERS checklist has 24 items that should ideally be present in the publications of studies on economic evaluation in health, but was not created to be an instrument of evaluation of methodological quality in addition. Both of them present flaws in the fact that they always relate clinical efficacy to new alternative interventions.

Therefore, the elaborated checklist contains 29 items (Table 5) which address the main points that characterize a health cost analysis. The initial objective of this checklist was to provide guidelines for the evaluation of articles submitted to the BMJ, enabling easier understanding of experts and non-specialist. This checklist is divided into three blocks of questions: i) drawing the study with 11 items; ii) data collection holding 9 items; And iii) analysis and interpretation of the results holding 9 items. The application of this quality instrument in this work was performed independently between the two reviewers. The discrepancies were solved by consensus and, in the absence of consensus, a third reviewer was consulted.

**Table 5:** Checklist economic evaluation.

Item	YES	%
**Drawing the Study**		
Research is adequate	22	100
The epidemiological source is stated	22	100
The study is identified as an economic evaluation	22	100
Provide a structured summary	13	60
Describe characteristics of population	13	60
Time horizon	11	50
Study perspective	17	80
The form of economic evaluation used is stated	14	64
The study was approved by an institution authorized in ethics in research	20	91
Conflicts of interest	12	55
Study funded	13	59
**Data Collection**		
The source(s) of costs estimates used are stated	19	86
The costs were clearly described	19	86
The valuation method is stated	12	55
Type of cost is stated	22	100
Currency, price date, and conversion	11	50
Unit costs are described in	11	50
The analytical model used is stated	11	50
Methods and assumptions for extrapolating results	11	50
The measurement of costs is adequate	18	81
**Analysis and Interpretation of the Results**		
Evidence of quality	18	81
Characterizing uncertainty	11	50
Outcome measures in health were clearly described, relevant to the study question	19	86
Ratio between health costs and outcomes	13	59
The approach to sensitivity analysis is given	13	59
Relevant aspects	11	50
The variation of costs over time is justified	13	59
Conclusions follow from the data reported	21	95
Conclusions are accompanied by the appropriate caveats	20	91

The checklist established three rating grades for the items: "Yes," "No," and "Not applicable." At the end of the classification, a relative value of each of these grades was settled for each study. The goal was to check the percentages of "Yes" for each question.

### Data analysis and interpretation

The profile of the studies and their characteristics were presented in tables, in order to allow comparison of the selected parameters, as well as the costs. This financial aspect was evaluated through the results in monetary amounts associated with the disease and the program.

The methodological variability of the monetary values limits the comparability of data, so a conversion of monetary values was performed in the concept of Purchasing Power Parity (PPP). PPP is an artificial currency also denominated "international dollars", which eliminates the differences of the countries and allows the income to be expressed in a common artificial currency [13].

All annual values reported in the studies were converted to the local currency at the exchange rate of the year of study. The value of the local currency has been inflated to 2015 with each country's Consumer Price Index (CPI), by http://fxtop.com/en/inflation.

After the values were corrected for inflation, the PPP was applied to the conversion rate in dollars PPP by http://data.worldbank.org/indicator/PA.NUS.PPP, to allow a greater comparison of the results. For studies that presented more than one annual value, an average was made between the years, always choosing the last year of the study to carry out the conversion (S2 Table).

## Results

**Fig 1:**
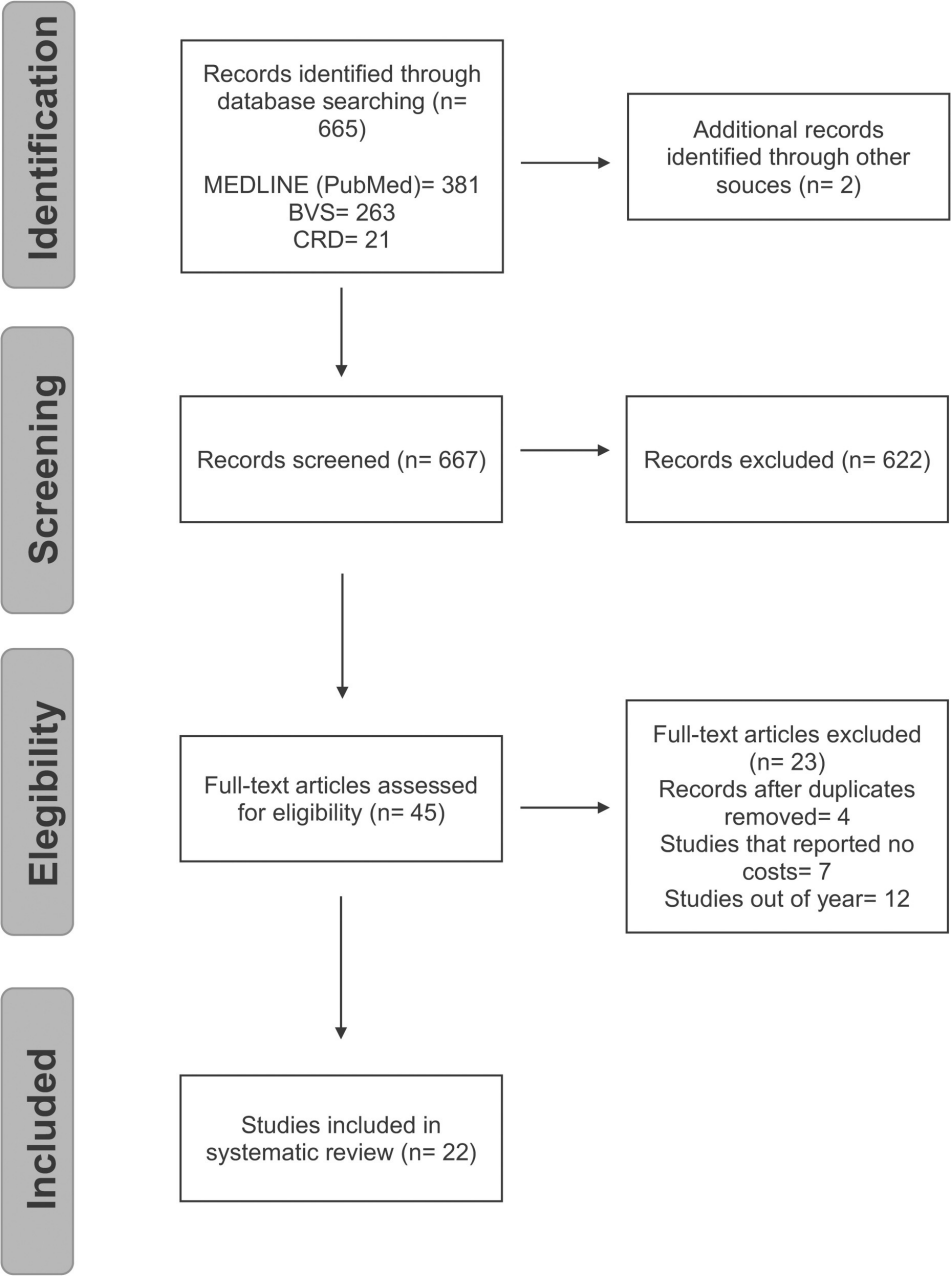
Flowchart of the selection of the studies included in the systematic review.

VHS: Virtual Health Library; CRD: Centre for Reviews and Dissemination.

The systematic review of costs of dengue, began in September 2015, in the Medline (via Pubmed), VHL, CRD databases, found 665 references. Of these, 56 were selected initially by titles and abstracts. After the removal of duplicates, 50 references remained. After analyzing full texts, 22 articles [14–35] were selected, according to the inclusion criteria previously established (Fig 1). The reason for exclusion in the last step has been declared (S1 Table).

The 22 studies were published as of 2005, of which seven (32%) were published in 2016. Only two studies (9%) analyzed only the cost of the program, fourteen studies (64%) only cost the disease, and six studies (27%) analyzed both costs (Table 2).

Twelve (55%) studies reported financial support, of which eight (36%) reported financial support from the Pharmaceutical Industry (Table 2).

After analyzing the general information of the selected studies, Table 2 goes to section A which shows information about study design, population included and comparators used (study methodology).

The time horizon of major analysis was 7 years [26], but only the last 3 years of this article were considered, the average time of analysis of the articles was of 1 year. Four (18%) studies have Brazil as the target population (Table 2).

The method for collecting cost data consisted of 10 studies (45%) of micro-costing and 12 (55%) of gross-costing (Table 3).

The cost components included in the economic evaluation were based on direct and indirect costs, of which 12 (55%) included both costs (Table 3).

The incidence of the disease in the period studied was considered in all studies (100%) (Table 3).

Twelve studies (55%) adopted the perspective of society, while ten studies (45%) used the perspective of the public health service provider, or a private budget holder (Table 4).

Four studies [18; 20; 24; 27] were not able to calculate the cost in dollars PPP in 2015, since they bring in their results costs of several localities of the world, making difficult to realize the inflation in the local currency. All four studies adopted perspective of society and their respective costs were US $ 1.8 billion in 2005 [27]; US $ 3.1 billion in 2007 [18]; US $ 40 billion in 2011 [24]; US $ 8.9 billion in 2013 [21].

In the comparison of monetary costs between the studies (Table 4), emerging countries as India had expenditures of 4.69 billion dollars PPP in direct medical costs (outpatient / hospital). Whereas Brazil spent 20.82 million dollars PPP in direct cost of prevention and control of *Aedes aegypti*. France, a developed country, spent 15 million dollars PPP on direct medical costs of hospitalization.

Two studies (9%) brought an analysis of intangible costs, represented by the Quality- Adjusted Life Years (QALY). In Panama [16] there was an average of 67% QALY during the worst days of illness in 2005, while in Malaysia [19] the average was 60% QALY in 2009. And three studies (14%) brought indirect cost analysis, represented by the Disability-Adjusted Life Years (DALYs). In Mexico [22] the annual disease rate averaged 65 DALYs per million inhabitants between 2010 and 2011. In Cambodia [28] the annual disease rate ranged from 24,3 to 100,6 DALYs per hundred thousand inhabitants between 2007 and 2008. The Americas presented an estimated 73,000 DALYs, with 131 DALYs per million inhabitants in 2004, the highest number per million inhabitants [18].

The performance of the studies in comparison to the checklist economic evaluation is satisfactory. All the studies contained more than 50% of the items required in total checklist (Table 5).

The most complete study reported 90% [22] of ‘Yes’ to the items present in the checklist, and the most incomplete study reported on the checklist of 29 items elaborated to evaluate the quality of economic evaluation studies 50% [14] of ‘Yes’.

From the items checked, in the first block that deals with the study design, all the studies are identified as an economic evaluation, but the structure of the abstracts in 9 studies was not appropriate, such as the presentation of objectives, perspective, methodology and results (Table 5).

In the second block, data collection was verified. The cost measurement is given in 86% of the studies, however, currency price readjustments for inflation or currency conversion, for the costs that were collected in different periods, were found in only 50% of the studies.

In the third block, data analysis and interpretation was determined, and 9 studies (41%) did not address sensitivity, while 11 studies (50%) did not correct methodological uncertainty, but all conclusions result from reported data, accompanied by 21 studies (95%) with appropriate warnings.

To sum up authors' conclusions regarding the cost found, costs of dengue are of great impact on the economy, and further studies are needed with a more accurate estimate for the decision makers. Therefore, carrying out this systematic review is of utter necessity.

## Discussion

### Methodological comparison between studies

Dengue is an acute illness and the incidence is its epidemiological measure [34], this factor is shown on all studies, leading to cost analysis over time and an evaluation of the effectiveness of intervention strategies, as they are based on cost of disease in a given year of study [36;37;38].

The studies included in the SR are recent, from 2005 on, which indicates that these studies of costs of dengue may be of growing interest for the inclusion of preventive interventions. One example is the dengue vaccine, which in recent years has arisen several candidates in pre-clinical and clinical developmental stages against the four serotypes of the dengue virus [39; 40] an example is Dengvaxia (CYD-TDV) from Sanofi Pasteur, the first live quadrivalent recombinant live vaccine registered in 2015 [41]. WHO recommends the inclusion of such intervention to countries that present epidemiological data indicating the economic burden of the disease [41], thus using these SR studies for cost-effectiveness calculations [38].

Amongst the identified studies, there was a great methodological variation of the costs found due to the influence of the methodological choices. In the 20 economic studies identified, they are directly related to dengue costs, but the method of costing and the composition of cost items differentiate from one study to the other, the form of micro-costing and gross-costing.

In the SR of Ernstsson and colleagues regarding the cost of multiple sclerosis disease, researchers also faced methodological differences in the inclusion of different types of costs [42]. It can be concluded that the calculation methods directly affect the comparability between the studies.

The time horizon of the economic evaluation of all the costs that are relevant to the desired results should be made explicit and justified in its methodology [43]. In twelve papers the time horizon was not made explicit, but that did not compromise the evidence of the data collected, since it was possible to obtain the period of data collection after the studies.

An analyze of the methodology used in these articles were identified specific questions relevant to the study of dengue costs, such as the definition of the study perspective, which can greatly cause variation in the results obtained [41]. The studies did not use only one perspective, but the perspective by the managing body as buyer of public and private health services, when approached from the perspective of the society most of the studies brought all the costs of the production of the service /procedure and the time wasted by the patients and their families, in addition to costs related to loss of productivity and premature death.

Thus, the economic analysis from the perspective of society brings an additional analysis, including not only an assessment of health costs, but also a measurement of the health consequences caused by dengue.

The measurement of these consequences can go beyond mortality and morbidity. The measurement of health-related quality of life, an evaluation that has increased significantly in the last 50 years, is recommended as the measurement of health outcome by international guidelines and by international health technology assessment agencies, such as NICE, the United Kingdom United [43].

In the articles, 1 [31] measured QALY, using EuroQol as a utility measure, and 7 articles [17;18;19;20;25;26;29] brought DALY, as an alternative to the use of QALY.

However, QALY is a broader and more complex indicator than DALY by incorporating quality of life beyond physical disability. The QALY and DALY values found were close to each other, unlike the analysis with the monetary value measurement [44].

The economic studies that bring in addition to COI, DALY and QALY, are the most appropriate studies for evaluating socially and financially viable public strategies [44].

### Comparability of cost between studies

Andersson proposed a methodology for comparing drug prices, and one of the criteria established by Andersson is to select countries with similar parameters and health system characteristics [45].

For the present work, as it deals with costs of the disease and the program that involves the use of medication or prophylactic measures, the analysis criteria used the health systems to evaluate countries that presented the highest cost, and the country of lowest cost.

Developing countries that had the highest costs with dengue [19;20;21;22;23;26], have common socioeconomic characteristics and similarities to health systems. Brazil and India, for example, have those similarities determined on their Constitution as an universal right [14;46;47]. India, being the second most populous country in the world, reported an annual average of 20,474 million cases of dengue fever, presenting the highest cost of the disease [14].

Argentina and Mexico presented similar costs, one of the main characteristics of both countries being the fragmentation of the health service systems, as well as its access to those services [48].

France as a developed country, presented the lowest expenditure. The French health service system was considered by the WHO close to being the best global healthcare, being largely financed by the government, the policy is centralized, further the state has the control of the activities of financial institutions, doctors and patients [49].

## Conclusion

Although there were methodological variations between the studies, the costs found within their perspectives analyzed in the included studies demonstrate that our results support that dengue has a great impact on the economy. The sum of dengue costs for the articles [14;15;16;17;19;20;22;23;25;26;28;29;30;31;32;33;34;35] showed that 18 countries generated a cost of approximately US$ 3.3 billion PPP in 2015.

### Strengths and limitations

The SR, was performed at all stages independently by two authors, possible disagreements of relevant studies were discussed among the authors.

It is the first review of costs of Dengue, in addition to using an approach that relates the studies to the local health system, indicates the methodological differences, points out what should be done in a study of economic analysis in health and analyzes the presence of factors in the studies.

The method chosen for recalculating costs, PPP dollars, made it easier to compare the results of study costs, since there was standardization of results in a single year, 2015.

Properly analyzing the results of cost evaluations using the resource allocation of decision is not an easy task because the studies have the interference of the time factor, the incorporation of new technologies and the local epidemiological scenario [50].

Research has shown that dengue imposes a significant level of financial burden on families and caregivers [15;16;17;18;20;21;22;23;24;25;26;27;28;30;31;33;34] but not all studies that brought the general aspects, for example, aspects that evaluate loss of work productivity, Family expenses (indirect costs).

Therefore, the total costs of dengue including all parameters of indirect costs would be much higher than those estimated in this RS.

A possible bias in the research is 40% of the studies being funded by the Sanofi industry, but the fact that a research receives financial support from an entity that has a direct interest in the subject being studied does not necessarily imply that researchers' conclusions are biased in all of these articles. It was presented the source of funding and passed the ethics committee in researches, being an acceptable practice and all studies funded by Sanofi have stated that there is no conflict of interest [51].

A survey including articles that use other types of analysis such as cost minimization may contribute to a more complete understanding of economic burden of Dengue [44].

## Supporting information

S1 Checklist This is the PRISMA 2009 checklist. (PDF)

S1 Table Reason for exclusion, based on the stated inclusion criteria. (TIF)

S2 Table Conversion ppp dollar. (TIF)
