# Supplementary figures and images for: Cost of illness and program of dengue: A systematic review
Source: PLoS One. 2019 Feb 20;14(2):e0211401. doi: 10.1371/journal.pone.0211401 (PMC6382265; doi:10.1371/journal.pone.0211401)

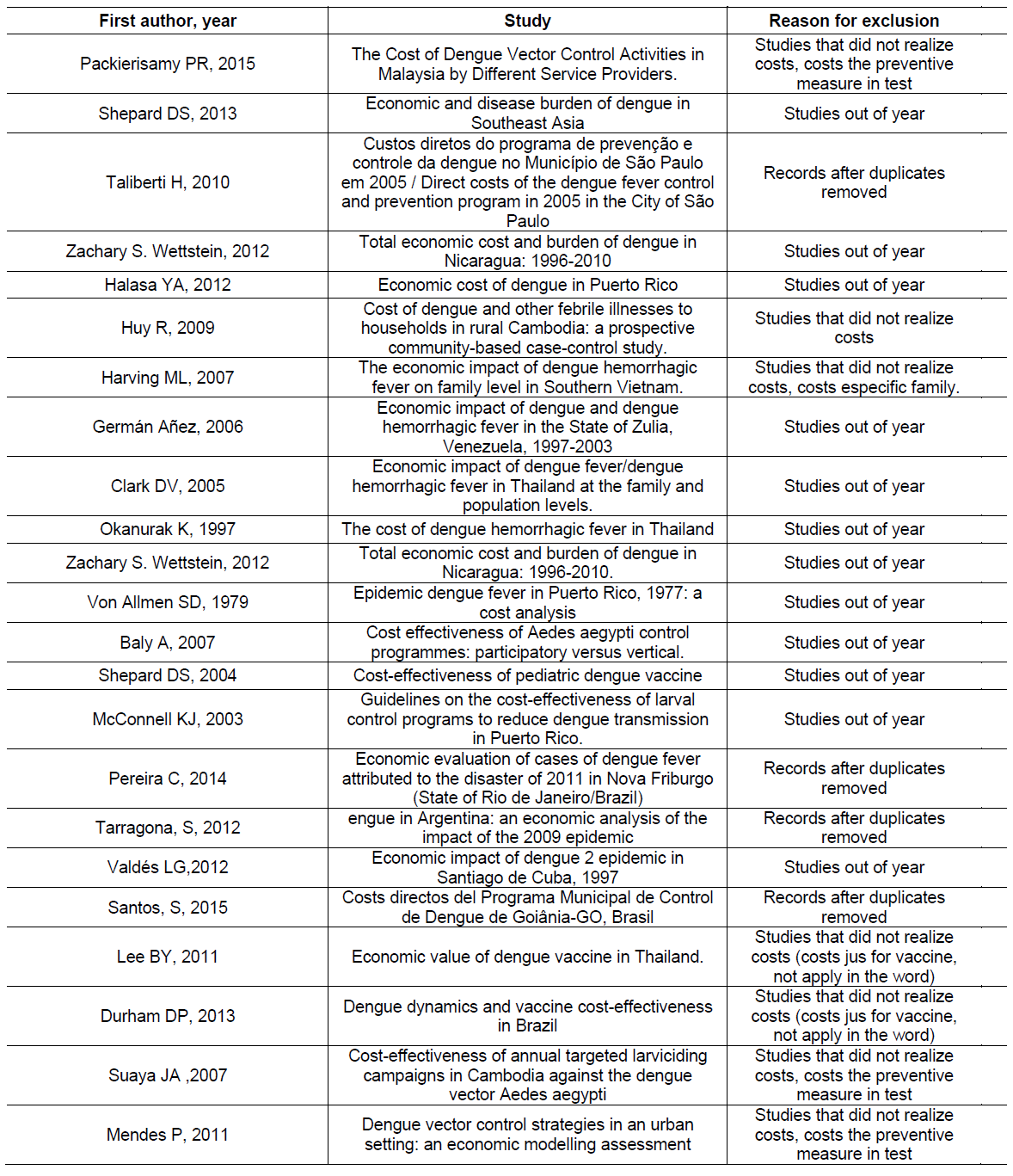

Supplement: S1 Table — (TIF) [file pone.0211401.s002.tif]

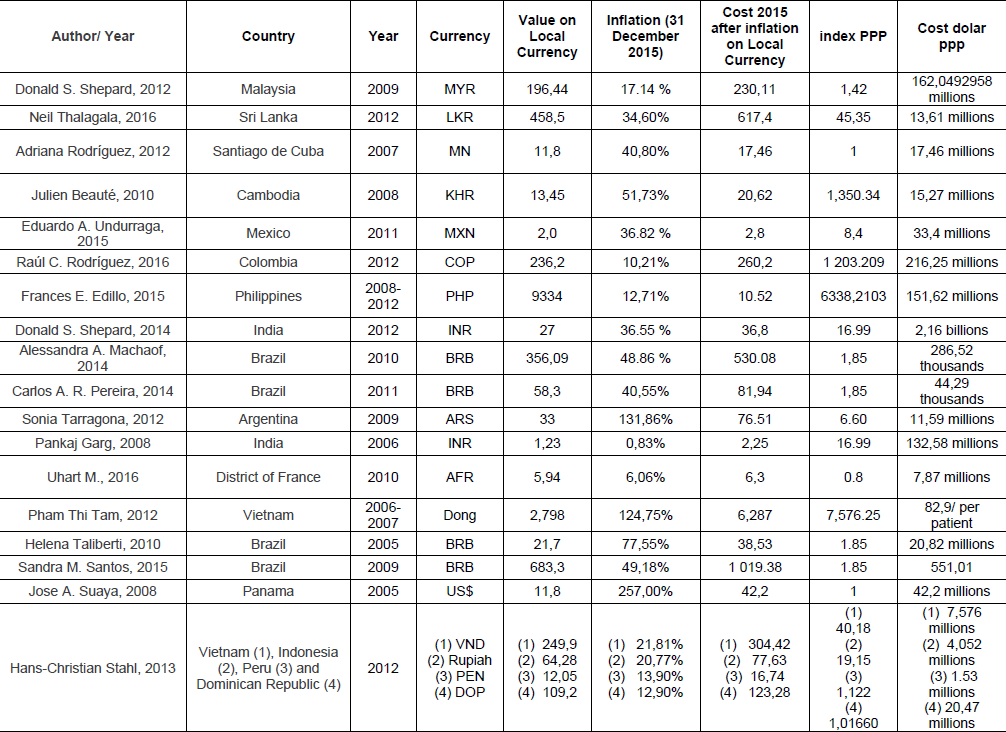

Supplement: S2 Table — (TIF) [file pone.0211401.s003.tif]
